# New York Medical and Surgical Society

**Published:** 1860-08

**Authors:** 


*New York Medical and Surgical Society.—Discussion on Diphtheria.*—Dr. C. M. Allin, of Flushing, related the histories of some cases of diphtheria which had lately come under his notice. About four weeks before he saw the first case, a child about six years of age was seized with an attack of well marked suppurative tonsilitis, which seemed to run its ordinary course for about a week or ten days, during which time an abscess formed and discharged; the swelling of the parts then began to subside. Two or three days subsequent to this, the child was suddenly seized with croupy symptoms. On examining the throat it was found that the swelling of the tonsils had returned, and at the location of the opening of the abscess there was discovered a large patch of false membrane, which covered the uvula, and extended down into the pharynx as far as could be seen. The child was very much prostrated, nearly pulseless, and was evidently rapidly sinking. The usual application of nitrate of silver to the parts, and the administration of stimulants was resorted to, but in vain, for the child died exhausted within twelve hours from the appearance of the first bad symptoms.

Three days after this, a younger child, in the same family, was attacked with sore throat, which presented the ordinary appearance of ulceration. In this case, however, none of the symptoms of prostration were present, neither did any diphtheritic membrane show itself. and the child recovered. Nothing more was seen of the disease for the next fortnight, when Dr. Bloodgood, the partner of Dr. Allin, was called to another case. He found the child very much in the condition of the first case, and learned that she had first complained of sore throat to her mother three or four days before. Various domestic remedies were resorted to, but the patient growing rapidly worse, Dr. B. was called in. On examination, the roof of the mouth, the throat, uvula, and all below the pharynx, as far as could be seen, was covered with a thick darkish yellow membrane. The countenance was very pale, and wore a very haggard expresssion; the pulse was very rapid and feeble, and there existed a marked croupy cough. Nothing, however, could save the child—it died the same evening. Early in the morning following, a child of the same family complained of sore throat. The tonsils and surrounding parts were congested. But nothing more was visible. A gargle of chlorate of potash was prescribed, and directions were left to feed up the patient well. On seeing the case again in the evening, he found an ulcerated spot about the size of a split pea on the left tonsil, to which he applied nitrate of silver. Chlorate of potash was then ordered internally, in addition to its use as a gargle. The next morning Dr. B. found that the ulcer referred to was larger than before, and there was also another of the same character on the tonsil of the opposite side. He applied the nitrate of silver again, and at the suggestion of Dr. Allin, hydrochloric acid was added to the mixture of chlorate of potash, in the proportion of a drachm of the former to two of the latter, in eight ounces of water: of this a teaspoonful was prescribed every two hours. I saw the case with him, continued Dr. A., a day or two after, and found that membrane had formed upon the surfaces of the ulcers referred to. The whole roof of the mouth was congested, but the membrane was confined to the uvula and parts immediately surrounding. The strength of the patient did not seem to be much impaired, the pulse being only 110, and we had strong hopes that the progress of the disease might be arrested. The next day, however, the child fell oft in strength, and we discontinued the potash mixture, ordering instead, the tincture of the sesquichloride of iron, to be used both as an internal remedy and a local application. At the time referred to, a portion of the membrane became detatched, and, on being removed by the forceps, was found to be very tough in consistence, very like the slough of a nitric acid issue in general appearance. Yesterday moruing (Friday) I called again to find the patient suffering from a croupy cough, while the surface of the throat covered by the membrane, had increased very much in extent. The child became more and more prostrated, and died at six o'clock the same evening—ten hours after the first symptoms of laryngeal trouble showed themselves. In neither of the two cases reported were post-mortem examinations made.

Dr. Allin stated that Dr. Vedder (of Flushing) had also met with this disease. One case occured in a child 18 months old, who sank rapidly and died in consequence of the appearance of croupy symptoms following an ordinary sore throat. The treatment consisted in the internal administration of the sesquichloride of iron and the local application of hydrochloric acid A post-mortem examination was made. The tongue, pharynx, and lining membrane of the œsophagus, down as far as the cardiac orifice of the stomach, was found covered with the characteristic membrane. It also formed a lining for the larynx and trachea, extending as far into the lungs as the minuter divisions of the bronchial tubes. The lungs aside from this, were only moderately congested. He stated that Dr. Vedder was treating, at that time, for diphtheria, a young girl 16 years of age, who was lying at the point of death. A blister was applied in one of Dr. Allin's cases, but the abraded surface was not covered with a diphtheritic membrane. In all the cases that recovered the convalescence was very much protracted.

Dr. A. C. Post referred to a case of this disease in a young woman, 21 years of age, which proved fatál in the course of the night in which she was attacked. Her child died a short time previous of the same disease. In both, the membrane made its first appearance upon the tonsils.

Dr. A. Clark had seen, since a year ago last autumn, somewhere between sixteen and twenty cases of diphtheria. The oldest case that he had seen prove fatal, was that of a lady, 22 years of age. The oldest person that he had seen affected with the disease was not over 36 years of age. But a small number of post-mortem examinations were made, but they were however sufficient to show a very great variety in the extent of the newly formed membrane. In some instances it extended throughout the pharynx, lining the larynx and trachea, and going down as far as the bronchial tubes could be conveniently opened, besides extending into the posterior nares. In one case this membrane could be seen from the front plunging up the nostrils. In other cases the larynx was not at all affected, the diseased action being confined to the pharynx and œsophagus. On the other hand, he had found the deposit confined to the larynx only. In some of the cases where no post-mortem examinations had been made, immense tubes or bands of thick leathery matter had been expectorated, but without being attended with any relief in the laryngeal symptoms, except in two instances, where recovery took place. In all the cases, so far as he had the means of knowing, the membrane was visable upon some portion of the fauces, most commonly upon one of the tonsils, before any symptoms of dyspnœa showed themselves, and before there were evidences of the formation of the deposit in any other part. In nearly one half the cases in which fatal results had occurred, such a state of things took place without dyspnœa, but with a set of symptoms such as he could hardly compare with those of any other disease. There was muscular force enough, yet there was a very marked feebleness of the pulse, which was attended with blueness of the nails and lips. He thought that it was a condition very apt to deceive a physician who saw such a case for the first time, and lead him to suppose that recovery might take place. In relation to the mode of invasion of this disease, Dr. C. stated that it had been exceedingly variable. I should think, continued he, that in the cases that I have seen, the severity of the symptoms of invasion have had some relation to the age of the patient, being more severe in those that are older. I do not, however, wish to make this a statement, it only is the result of a limited observation. In some children the ordinary symptoms of sore throat first present themselves, the membrane forms slowly, but the issue in such cases is hardly less fatal than that of others. In other instances the invasion is very brisk, the patient has two or three chills in the course of the day, while in the more insidious forms referred to the duration is a fortnight including the early illness. In those cases that recovered the convalescence was very much protracted. In answer to a question from Dr. Post, he stated that he recollected one case that lasted but three and a half days.

Dr. McCready next cited the following case:—A patient of his, a child was first seized with the ordinary symptoms of sore throat. In the course of a day or two membrane showed itself upon the tonsils, but soon disappeared entirely, and everything pointed towards a recovery. After the lapse of about a week, however, membrane appeared in the nostrils, when the child became suddenly collapsed and died within twenty-four hours after. In that case it seemed that the disease disappeared from the tonsils and afterwards selected the nostrils as its seat.

Dr. Clark stated that in one case he saw with Dr. Crane, death took place in a somewhat similar way. All the membranes had been discharged and the boy was regarded as fairly convalescent. I visited the case one morning about ten days after the severe symptoms and thought him doing well. He was able to sit up a considerable portion of the day; his strength was increasing, and his friends were encouraged. About two o'clock of the same day, Dr. Crane was sent for, and found the child pale and sinking; the pulse at times would be scarcely perceptible, then it would become more full, but the exhaustion was so extreme that the slightest movement, even raising the head, would bring on a fainting fit. I arrived in time to see the child breathe his last. His appearance at the time I saw him was that of a person dying from internal hemorrhage, and the history of the fatal attack tended to strengthen the suspicion. No autopsy could be obtained. In regard to treatment, Dr. Clark stated that when he first met the disease last autumn, the treatment was very varied and unsettled, and he was not satisfied with any method then in use. Seeing a statement that the Doublin and Edinburgh physicians were disposed to rely upon the muriated tincture of iron, he began to advise that remedy. He had since fallen into the practice, now generally adopted here, viz: sustaining the patient by quinia, given freely the muriated tincture of iron, wine, &c., and interfering but little with the membrane. He did not favor the use of mercurials on account of their constitutional effect. Bretonneau used them at first, but was forced to discontinue them for this reason. In reply to the question, whether he regarded diphtheria a different disease from the croup, Dr. Clark said that he did; one difference was the frequent occurrence of an abundant exudation in the substance and upon the surface of the membrane, and then the appearance of the membrane itself, the border of the patch being surrounded by an intensely red margin, giving it the appearance of a slough about to separate.

Dr. Buck said he had seen patients die even after the separation of the membrane. In reference to treatment, he stated that Dr. Lindsley's great reliance in these cases was mercurial fumigations. He had seen recoveries under its use, and in one, particularly, it was continued day and night for eight days. The disease seemed to be kept in check during its use, but any cessation in its application was followed by an aggravation of the most unpleasant symptoms, and it was not until the eighth day that the relief obtained was permanent. The convalescence was gradual and protracted. In this case the exudation on the tonsils was recognized at the first visit, and within twenty-four hours after hoarseness and laryngeal symptoms appeared. He was so favorably impressed with the value of his remedy that he advises its thorough trial. The fumigation was affected by enveloping the child's head with a blanket, and then heating an iron body to a red heat, throwing upon it cinnabar, when the whole was passed under the blanket. When the child was very small it was necessary that the attendant should also be subjected to the fumigation.

Dr. McCready said that he had seen a case with Drs. Parker and Van Buren which was successfully treated by the method of fumigation.

Dr. Jas. R. Wood remarked that Dr. Lindsley had used the cinnabar in fumigation in croup for many years. He had himself tested its efficacy and could report favorably. Diphtheria, he continued, is a different disease from inflammatory croup, being attended with more nervous prostrations, and the patient running rapidly into a typhoid condition. It is essentially a blood disease. Again they differ in the location of the exudation; in true croup it does not always commence upon the fauces and extend unto the larynx; but in diphtheria he had always first discovered the exudation in the fauces or upon the tonsils, and the laryngeal symptoms supervened soon after.

